# Dry eye symptoms and impact on vision-related function across International Task Force guidelines severity levels in the United States

**DOI:** 10.1186/s12886-018-0919-7

**Published:** 2018-09-29

**Authors:** Laurie Barber, Omid Khodai, Thomas Croley, Christopher Lievens, Stephen Montaquila, Jillian Ziemanski, Melissa McCart, Orsolya Lunacsek, Caroline Burk, Vaishali Patel

**Affiliations:** 1 Little Rock Eye Clinic, 203 Executive Court, Suite A, Little Rock, AK 72205 USA; 2 Mobile Medical Solutions, Inc., Foothill Ranch, CA USA; 3 Central Florida Eye Institute, Ocala, FL USA; 4 Southern College of Optometry, Memphis, TN USA; 5 West Bay Eye Associates, Warwick, RI USA; 6 School of Optometry, University of Alabama at Birmingham, Birmingham, AL USA; 7 Xcenda, Palm Harbor, FL USA; 8 Health Outcomes Consultant, Laguna Beach, CA USA; 9 Allergan plc, Irvine, CA USA

**Keywords:** Dry eye disease, Ocular Surface Disease Index, International Task Force guidelines, Vision-related function

## Abstract

**Background:**

International Task Force (ITF) guidelines established a grading scheme to support treatment of dry eye disease based on clinical signs and symptoms. The purpose of this study was to assess the impact of dry eye on vision-related function across ITF severity levels using the Ocular Surface Disease Index (OSDI) questionnaire.

**Methods:**

Non-interventional, cross-sectional study of prescription treatment-naïve dry eye patients seeking symptom relief at 10 ophthalmology and optometry practices. Clinicians assessed corneal and conjunctival staining, tear break-up time, Schirmer’s test (type I with anesthesia), and best-corrected visual acuity. Patients completed the OSDI questionnaire and OSDI overall and domain (Symptoms, Visual Function, and Environmental Triggers) scores were compared across ITF guidelines severity levels (1–4).

**Results:**

Of 158 patients (mean age, 55 years) enrolled, 52 (33%) were ITF level 1, 54 (34%) ITF level 2, and 52 (33%) ITF levels 3/4 combined. No significant differences were observed in most baseline characteristics. Overall OSDI scores (mean [standard deviation]) were 26.5 [20.0] for ITF level 1, 33.8 [17.5] for ITF level 2, and 44.9 [26.1] for ITF level 3/4 cohorts (*P* < 0.0001). Component OSDI Symptoms, Visual Function, and Environmental Triggers domain scores all worsened with increasing ITF severity level (*P* ≤ 0.01).

**Conclusions:**

Dry eye disease has significant deleterious impact on vision-related function across all ITF severity levels.

## Background

Dry eye is a multifactorial disease of the ocular surface resulting in discomfort, visual disturbance, and instability of the tear film [1]. In the early 2000s, it was estimated that dry eye affected over 7 million people over the age of 40 years in the United States [2, 3]. Prevalence has likely increased significantly over the past 10 years with the escalation of risk factors such as an aging population, a greater number of refractive laser surgeries, and more frequent use of contact lenses, computers, smartphones, and tablets [2–5]. Additionally, women are known to experience dry eye more frequently than men, potentially owing to hormone fluctuations during the menstrual cycle or menopause, and from use of oral contraceptives or hormone replacement therapy [6].

The symptoms that commonly compel patients with dry eye to seek treatment from ophthalmologists and optometrists include ocular discomfort and irritation, burning, itching, and blurred vision [7, 8]. In addition to blurring of vision, other changes in visual function noted in dry eye patients include reductions in functional visual acuity [9] and contrast sensitivity [10], optical aberration due to tear film irregularity [11], and degradation of retinal image quality [12]. Dry eye disease significantly affects patients’ visual function and greatly impacts social and physical functioning, workplace productivity, and quality of life [13, 14]. For example, ocular discomfort and dryness are often reported as the primary reason for discontinuation of contact lens wear [15–18], negatively affecting patients’ quality of life. In one study conducted in the United Kingdom using utility assessment (Time Trade-Off and Standard Gamble methods) to quantify and understand the impact of a given health condition relative to other diseases, severe dry eye utilities were similar to those associated with dialysis or severe angina [19].

Symptomatic dry eye disease can present without evidence of ocular surface damage or changes in tear flow [13, 14, 20]. Poor correlation has been found between symptoms and clinical measures of dry eye disease [21–24], and patient-reported dry eye symptoms have been demonstrated to be more reproducible from visit to visit than many of the clinical signs used to diagnose and monitor dry eye [25]. Consequently, quality of life or patient-reported outcomes (PRO) evaluations have been used to provide clinicians with valuable information on the impact of dry eye disease and the effectiveness of treatment [26, 27]. The Ocular Surface Disease Index (OSDI) is a validated PRO instrument that provides a measure of the ocular symptoms and disability associated with dry eye [20, 28]. The OSDI was developed as a brief, self-administered questionnaire to provide rapid evaluation of the range and frequency of ocular symptoms associated with dry eye disease, and their impact on patients’ visual functioning. The questionnaire includes 3 subscales, which cover ocular discomfort, limitations in performance of daily activities affected by dry eye, and the susceptibility of dry eye symptoms to environmental factors. As an instrument, OSDI shows internal consistency, with good to excellent test-retest reliability and excellent discriminant validity for measuring dry eye symptoms [28].

The International Task Force (ITF) guidelines for diagnosis and treatment of dry eye were established by an expert panel that considered dry eye disease severity to be the most important factor in making treatment decisions [29]. The ITF panel established a 4-level dry eye severity grading scheme based on signs and symptoms [29]. A study evaluating the implementation of this scheme by clinicians found these guidelines to be simple and efficient for assessing dry eye severity and for supporting treatment decisions [30]. Notably, use of the ITF guidelines led clinicians to focus on patient symptoms and initiating early treatment rather than relying on dry eye diagnostic tests [30].

As standard clinical measures of dry eye provide only a partial picture of the disease experience, it is difficult to appreciate how dry eye is perceived by the patient. The current study was conducted to quantify the frequency of dry eye symptoms and their impact on vision-dependent functioning across different ITF levels, using the OSDI questionnaire. Patients were prescription treatment-naïve (received no prescription therapy for dry eye disease) presenting to their clinicians with complaints of dry eye. Although studies have assessed symptoms in patients with dry eye [7, 13, 14, 20], to date no real-world, clinic-based studies assessing dry eye symptoms and the impact of disease based on ITF severity categories have been reported in the published literature.

## Methods

### Study design and patient selection

This was a non-interventional, cross-sectional study conducted from July 2014 to October 2014. To ensure real-world representation of dry eye patients’ access to care, 5 ophthalmology and 5 optometry clinical practices across the United States were recruited to enroll patients. Prescription treatment-naïve dry eye patients at least 18 years of age, seeking routine consultation for relief of dry eye symptoms, were enrolled consecutively. For study eligibility, patients were required to show clinical signs of dry eye, as assessed from conjunctival (lissamine green) and corneal (fluorescein staining and Schirmer’s test (type I with anesthesia), during screening. The study excluded patients with prior use of prescription dry eye medication or punctal plugs; ocular surgery within the previous 6 months; use of antibiotics, corticosteroids, immunosuppressant medications, topical nonsteroidal anti-inflammatory drugs or antivirals within 30 days of the start of the study; a diagnosis of active ocular allergies; infection of the anterior segment or uveitis; and a systemic or ocular disorder or condition deemed by the investigator to potentially affect interpretation of study results. The study protocol was reviewed and approved by the following independent review boards: Liberty Institutional Review Board (DeLand, FL), Southern College of Optometry Institutional Review Board (Memphis, TN), and Western Institutional Review Board (Puyallup, WA). Patients provided written informed consent prior to study participation.

### Data collection and study measurements

During the enrollment visit, patient demographics as well as medical and medication histories were recorded for ocular and nonocular conditions. Clinicians conducted an examination of the worse eye (as reported by the patient) or the right eye if both eyes were reported to be equally affected. Clinicians were selected on the basis of their expertise in ocular surface disease and were instructed to use clinical standard of care in their grading of ocular surface staining. For corneal punctate staining with fluorescein, the entire cornea was examined using slit-lamp evaluation with a yellow barrier filter and cobalt blue illumination, and staining was graded as “none”, “mild”, “marked”, “severe”, as well as “central” or “non-central”. For conjunctival staining with lissamine green, interpalpebral staining was measured between 30 s and 2 min after instillation of the dye and likewise graded according to the clinician’s judgement as “none”, “mild”, “marked”, or “severe”. Tear break-up time (TBUT), the time (seconds) until random location tear break-up between blinks, and the amount of wetting (mm) on Schirmer’s test (type I with anesthesia) performed for 5 min also were assessed. Since the study was performed within the setting of routine clinical practice, best-corrected visual acuity was evaluated (both eyes) using Snellen notation. No safety assessment was performed, as there was no intervention in this study.

Clinicians’ assessments of dry eye severity were based on objective measures and patient-reported visual symptoms of disease. In accordance with ITF guidelines, disease severity in the study eye was graded on a 4-point scale ranging from level 1 (mild-to-moderate symptoms plus mild-to-moderate conjunctival signs), via level 2 (moderate-to-severe symptoms plus either tear film signs, conjunctival staining, mild corneal punctate staining, or visual signs) and level 3 (severe symptoms plus either marked corneal punctate staining, central corneal staining, or filamentary keratitis), to level 4 (severe symptoms plus either severe corneal staining, with erosions or conjunctival scarring) [29]. Dry eye symptoms identified by the ITF panel as being of particular relevance in determining disease severity are ocular discomfort (itchiness, burning, foreign body sensation, and sensitivity to light) and visual disturbance [29]. Stratification by ITF severity was not disclosed to patients to ensure unbiased completion of survey questionnaires following their clinical examination.

The OSDI questionnaire was used to quantify the symptomatic and functional impact of dry eye, as perceived by the patient. The questionnaire consists of 12 items (questions) included in 3 subscale domains measuring the frequency of (1) ocular symptoms (specifically sensitivity to light, grittiness, sore/painful eyes, blurred vision, and poor vision) (questions 1–5), (2) visual problems impacting daily activities (reading, television viewing, computer work, and night-time driving) (questions 6–9), and (3) ocular discomfort triggered by environmental factors (wind, low humidity, and air conditioning) (questions 10–12) over the previous week. The response to each question is graded on an analog scale of 0 (none of the time), 1 (some of the time), 2 (half of the time), 3 (most of the time), and 4 (all the time). Overall OSDI and subscale domain scores range from 0 to 100, with higher scores representing greater ocular disability, and based on the overall score, patients can be classified as mild (13–22), moderate (23–32), or severe (≥33) [31].

Patient and Clinician Assessment Forms were used to record patient data and results of diagnostic and survey tests. Prior to study initiation, 2 clinicians took part in 60-min interviews and 3 patients participated in 30-min interviews to ensure that the survey’s wording and directions were clear and easily understood. The final Clinician Assessment Form and Patient Assessment Form were slightly revised as a result.

### Study endpoints and data analysis

Study endpoints were clinicians’ assessments of dry eye signs (ocular surface staining intensity, TBUT, and Schirmer score) as well as OSDI overall and Symptoms, Visual Function, and Environmental Triggers domain scores, categorized by ITF severity level.

Data were summarized using descriptive statistics for continuous and categorical variables. Frequencies reported for individual OSDI questions were collapsed into 2 groups according to how often each item was reported to have occurred: less than half of the time versus at least half of the time. Comparisons between ITF severity levels for demographics, clinical characteristics, and diagnostic tests were performed using 1-way analysis of variance (means) and chi-square test (proportions). The mean overall and subscale domain OSDI scores were compared across ITF severity levels using general linear models adjusted for age, gender, and hypertension. The percent of patients responding to individual OSDI questions among ITF severity levels was compared using the chi-square test. Statistical significance was set at *P* < 0.05.

Sample size estimates for analysis of variance of OSDI scores across ITF severity levels were based on previous reports of a minimal clinically important difference in overall OSDI score of approximately 5 points for patients with mild-to-moderate dry eye and 10 points for those with severe dry eye, where the standard deviation (SD) for the minimal clinically important difference in OSDI score ranged from 2.5 to 23.1 points [28, 31]. A sample size of at least 150 patients was determined to have > 90% power to detect a minimal clinically important difference of at least 5 points when the SD was 13 or less, assuming α = 0.05 for a 1-way analysis of variance. For analysis, patients were grouped into 3 cohorts based on ITF severity level, with an estimated 50 patients in ITF level 1, 50 patients in ITF level 2, and 50 patients in ITF level 3/4 combined. It was anticipated that there would be fewer treatment-naïve patients in the more severe ITF levels who would meet study eligibility criteria, making it difficult to enroll 50 patients in each of the ITF level 3 and 4 categories. The F test statistic threshold to reject the null hypothesis of no difference in OSDI scores between the 3 ITF severity levels was 2.662. Power calculations based on a planned sample size of 150 patients indicated that 1-way analysis of variance would have 80% power to detect an effect size of 0.26 at α = 0.05.

## Results

### Patient demographics and clinical characteristics

A total of 158 patients were recruited for the study; 52 (33%) were categorized in ITF level 1, 54 (34%) in ITF level 2, and 52 (33%) in ITF level 3/4 combined (of which 39 [25%] were in ITF level 3 and 13 [8%] were in ITF level 4). The study population had a mean age of 55 years and was predominantly female (82%) and white (86%); 76% of patients had college education or higher, and 65% were employed at the time of the study (Table 1). There were no differences in patient demographics, including education, employment status, geographic location, household income, or health care coverage, between the 3 ITF severity level cohorts. Additionally, no significant differences in concomitant non-dry eye-related ocular and non-ocular comorbidities were observed across the 3 ITF cohorts, except for hypertension (*P* < 0.01). Individual comparisons between ITF severity levels revealed differences in hypertension, diabetes and hyperlipidemia, which were significantly more frequent (*P* < 0.05) in the ITF level 3/4 cohort compared with ITF level 1 or ITF level 2 (Table 1).

**Table 1:** Patients’ demographic and clinical characteristics by ITF severity level

Baseline characteristic	Overall (*N* = 158)	Dry eye severity level		
		ITF 1 (*n* = 52)	ITF 2 (*n* = 54)	ITF 3/4 (*n* = 52)
Age, mean (SD), y	55 (16)	53 (13)	57 (17)	56 (17)
Female, *n* (%)	130 (82)	43 (83)	41 (76)	46 (88)
White, *n* (%)	136 (86)	41 (79)	47 (87)	48 (92)
Geographic region, *n* (%)				
Midwest	7 (4)	3 (6)	4 (7)	0
Northeast	37 (23)	12 (23)	12 (22)	13 (25)
South	80 (51)	28 (54)	28 (52)	24 (46)
West	34 (22)	9 (17)	0 (19)	15 (29)
Education level, *n* (%)				
High school or less	38 (24)	12 (23)	12 (22)	14 (27)
Some college or higher	120 (76)	40 (77)	42 (78)	38 (73)
Employment status, *n* (%)				
Employed	103 (65)	36 (69)	36 (67)	31 (60)
Retired/disabled	47 (30)	12 (23)	16 (30)	19 (37)
Nonemployed	8 (5)	4 (8)	2 (4)	2 (4)
Ocular comorbidity, *n* (%)				
Cataract	48 (30)	13 (25)	16 (30)	19 (37)
Primary open-angle glaucoma	8 (5)	3 (6)	2 (4)	3 (6)
Ocular hypertension	2 (1)	0	0	2 (4)
Sjögren’s syndrome	4 (3)	1 (2)	1 (2)	2 (4)
Ocular allergy	2 (1)	2 (4)	0	0
Nonocular comorbidity, *n* (%)				
Hypertension	55 (35)	12 (23)*	16 (30)*	27 (52)
Hyperlipidemia	37 (23)	10 (19)	9 (17)*	18 (35)
Gastrointestinal disorders^a^	20 (13)	5 (12)	16 (11)	9 (17)
Diabetes	17 (11)	3 (6)*	4 (7)	10 (19)
Rheumatologic disease	14 (9)	3 (6)	3 (6)	8 (15)
Asthma	11 (7)	1 (2)	7 (13)	3 (6)
Peripheral vascular disease	2 (1)	1 (2)	1 (2)	0
Congestive heart failure	2 (1)	0	1 (2)	1 (2)
Angina	2 (1)	0	0	2 (4)
Other	26 (16)	7 (13)	13 (24)	6 (12)

*P < 0.05 versus ITF 3/4 by chi-square test

^a^Gastroesophageal reflux disease or peptic ulcer disease

*ITF* International Task Force, *SD* standard deviation

### Clinical assessments

Table 2 summarizes the intensity of ocular surface staining and tear film signs for each ITF level. Central corneal staining, which is indicative of more severe dry eye [29], was present in 56% of patients in ITF level 3/4 but no more than 2% of patients in either ITF level 1 or ITF level 2 (*P* < 0.0001 across all ITF levels). In addition, the mean grade of both corneal and conjunctival staining rose as ITF level increased (*P* < 0.0001 across all ITF levels). TBUT decreased significantly as ITF severity increased from level 1 (mean [SD], 8.4 [3.6] s) to level 3/4 (3.7 [2.0] s; *P* < 0.0001 across all ITF levels). In addition, Schirmer’s test scores were significantly lower in ITF level 3/4 (mean [SD], 7.2 [5.6] mm) compared with ITF level 1 (mean [SD], 13.6 [7.9] mm; *P* < 0.0001 across all ITF levels). The proportion of study eyes with visual acuity better than 20/40 declined numerically as ITF severity increased, while the proportion of eyes with visual acuity of 20/40 or worse rose numerically with increasing ITF severity (Table 2).

**Table 2:** Clinical assessment of dry eye by ITF severity level

Dry eye assessment	Overall (*N* = 158)	Dry eye severity level		
		ITF 1 (*n* = 52)	ITF 2 (*n* = 54)	ITF 3/4 (*n* = 52)
Left eye visual acuity, *n* (%)				
< 20/20	11 (7)	4 (8)	5 (9)	2 (4)
20/20 to < 20/40	134 (85)	46 (88)	45 (83)	43 (83)
20/40 to 20/60+	13 (8)	2 (4)	4 (7)	7 (14)
Right eye visual acuity, *n* (%)				
< 20/20	12 (8)	3 (6)	6 (11)	3 (6)
20/20 to < 20/40	133 (84)	47 (90)	45 (83)	41 (79)
20/40 to 20/60+	13 (9)	2 (4)	3 (6)	8 (15)
Central corneal staining, *n* (%)	30 (19)	0^†^	1 (2)^†^	29 (56)
Corneal staining, mean (SD)	1.2 (0.8)	0.7 (0.5)*^,†^	1.2 (0.6)^†^	2.3 (0.5)
Conjunctival staining, mean (SD)	1.3 (0.8)	0.8 (0.5)*^,†^	1.2 (0.7)^†^	2.0 (0.8)
TBUT, mean (SD), s	6.2 (4.3)	8.4 (3.6)*^,†^	6.4 (5.3)^†^	3.7 (2.0)
Schirmer’s test type I, mean (SD)	10.3 (7.0)	13.6 (7.9)*^,†^	10.3 (6.0)^†^	7.2 (5.6)

*P ≤ 0.01 versus ITF 2; ^†^*P* ≤ 0.01 versus ITF 3/4 by 1-way analysis of variance (means) and chi-square test (proportions)

*ITF* International Task Force, *SD* standard deviation, *TBUT* tear break-up time

### OSDI questionnaire scores by ITF severity level

Analysis of the OSDI questionnaire responses revealed that as ITF severity level increased, patients had worse OSDI overall and Symptoms, Visual Function, and Environmental Triggers domain scores (Table 3). Overall OSDI scores (mean [SD]) were 26.5 [20.0], 33.8 [17.5], and 44.9 [26.1] for ITF level 1, ITF level 2, and ITF level 3/4 cohorts, respectively (*P* < 0.0001 across all ITF levels). As expected, OSDI Symptoms domain score increased, indicating greater frequency of symptoms, as ITF severity level increased (*P* < 0.0001 across all ITF levels). However, OSDI Visual Function domain score and Environmental Triggers domain score also increased, suggesting more frequent disability, as ITF level increased (*P* = 0.0013 and *P* = 0.0107, respectively, across all ITF levels). Comparison of OSDI overall and subscale domain scores between individual ITF severity levels indicated that patients in ITF level 3/4 had significantly higher scores than those in ITF level 1 (*P* ≤ 0.005) and ITF level 2 (*P* ≤ 0.03, except for the Environmental Triggers domain). Patients in ITF level 2 also had significantly higher OSDI overall and Symptoms subscale domain scores compared with ITF level 1 (*P* ≤ 0.04) (Table 3).

**Table 3:** OSDI questionnaire scores

OSDI score, mean (SD)		Dry eye severity level			*P* value^a^
	Overall (*N* = 158)	ITF 1 (*n* = 52)	ITF 2 (*n* = 54)	ITF 3/4 (*n* = 52)	
Overall	35.1 (22.6)	26.5 (20.0)*^,†^	33.8 (17.5)^‡^	44.9 (26.1)	< 0.0001
Symptoms domain	32.8 (22.2)	23.3 (18.1)*^,†^	32.3 (17.2)^‡^	42.7 (26.2)	< 0.0001
Visual-Related Function domain	32.8 (25.1)	26.8 (23.0)^†^	28.6 (21.0)^‡^	42.7 (28.2)	0.0013
Environmental Triggers domain	43.3 (30.1)	34.2 (27.7)^†^	43.2 (29.1)	52.2 (33.0)	0.0107

*P < 0.05 compared with ITF level 2; ^†^*P* ≤ 0.005 compared with ITF level 3/4; ^‡^*P* < 0.05 compared with ITF level 3/4

^a^Comparison of scores across all ITF levels using general linear models adjusted for gender, ethnicity (overall, Symptoms domain), and hypertension

*ITF* International Task Force, *OSDI* Ocular Surface Disease Index, *SD* standard deviation

**Fig. 1:**
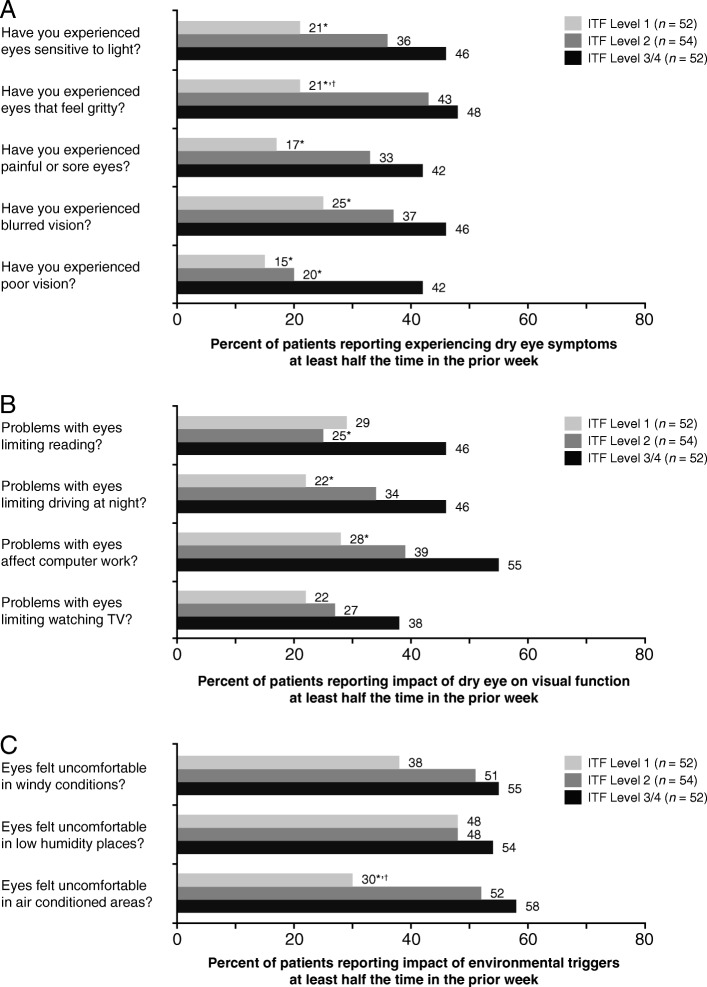
Proportion of patients indicating on OSDI questionnaire components that at least half of the time in the past week: (**a**) experienced dry eye symptoms (questions 1–5); (**b**) had problems with their eyes limiting visual function (questions 6–9); and (**c**) had eyes that felt uncomfortable in certain environmental conditions (questions 10–12) by ITF dry eye severity level. *P < 0.05 compared with ITF level 3/4; ^†^*P* < 0.05 compared with ITF level 2 by chi-square test; sample size varied for visual function and environmental triggers, as not all patients performed tasks over the previous week. *OSDI* Ocular Surface Disease Index, *ITF* International Task Force, *TV* television

Generally, a greater percentage of patients in ITF level 2 and ITF level 3/4 experienced frequent (at least half of the time) symptoms of light sensitivity, eye grittiness, painful/sore eyes, blurred vision and poor vision (Fig. 1a) and frequent dry-eye-related impairment of reading, night-time driving, computer work, and watching television (Fig. 1b) than patients in ITF level 1. The percentage of patients who reported frequent eye discomfort in windy and low humidity conditions was generally similar across ITF severity levels; however, the proportion of patients who reported frequent eye discomfort in air-conditioned areas was higher in ITF levels 2 and 3/4 compared with ITF level 1 (Fig. 1c).

## Discussion

Variation in diagnostic criteria and clinical measures of dry eye disease and its severity have been reported [25], and poor correlation between clinical signs and patient-reported symptoms has been documented in previously published literature [21–23]. Discordance between ocular surface findings and symptoms of dry eye may occur in the early stages of disease where symptoms can exist without clinical signs of dry eye [32]. Some investigators have hypothesized that chronic dry eye symptoms occurring in the absence of clinical signs of dry eye might be of neuropathic origin, arising as a result of central sensitization and manifesting as neuropathic ocular pain [33, 34]. The ITF guidelines were developed from expert consensus to establish severity criteria and treatment recommendations for dry eye [29], and formed a foundation for the 2007 International Dry Eye Workshop management and therapy of dry eye guidelines [35]. To date, there are no studies that have evaluated the impact of disease, in particular its functional impact, in prescription-naïve dry eye patients across ITF severity levels in a real-world, clinic-based setting.

In this observational study of patients with symptomatic dry eye, no significant overall differences were observed in patient demographics, concomitant non-dry eye-related ocular conditions, and comorbidities (other than hypertension) across the 3 dry eye patient cohorts based on ITF severity level (ITF level 1, ITF level 2, and ITF level 3/4 combined). Almost one-third of the patients enrolled in the study had cataracts and less than 10% had glaucoma. Very few (3%) patients enrolled in this study had Sjögren’s syndrome, an autoimmune condition associated with severe dry eye. Of the 30 patients enrolled who presented with central corneal staining, 29 were in ITF severity level 3/4. This, together with the higher prevalence of cataract in ITF severity level 3/4, may have contributed to the greater proportion (≥2 times) of patients in the ITF level 3/4 cohort who reported poorer visual acuity (20/40 to 20/60+) in both eyes compared with the lower ITF severity level 1 and 2 cohorts.

The study demonstrated that all OSDI subscale scores, including those for Visual Function and Environmental Triggers, were significantly worse in higher ITF severity level cohorts. This would indicate that the observed deterioration in OSDI overall score with increasing ITF severity was not driven solely by worsening dry eye symptoms. Pairwise tests demonstrated that overall and subscale domain OSDI scores were significantly higher in the ITF level 3/4 cohort compared with ITF level 1 and ITF level 2 cohorts, except for the Environmental Triggers domain score, which was not significantly different between ITF level 3/4 and ITF level 2 cohorts. In addition, analysis of individual OSDI questions demonstrated that a higher percentage of ITF level 3/4 patients experienced not only more frequent symptoms of dry eye, but also more frequent disruption of daily tasks and more frequent symptoms from environmental triggers of dry eye. Assessed against the published OSDI guideline categories of mild (13–22), moderate (23–32), and severe (≥33) ocular surface disease [31], patients in this study generally had frequent ocular disability, as reflected in overall mean OSDI scores of 26.5, 33.8, and 44.9 in ITF level 1, 2, and 3/4 cohorts, respectively, although intracohort variation in overall OSDI score was appreciable (based on standard deviation values in Table 3). Observed differences from the published guidelines may be associated with a different anchor (such as Global Clinician’s Assessment versus ITF guidelines) that was used to establish disease severity [31].

Our study results, obtained from a sample of prescription treatment-naïve patients presenting with symptomatic dry eye in the real-world setting, are consistent with previously reported clinical study findings that OSDI overall scores increase as levels of dry eye severity worsen. In a study by Schiffman et al. [28], patients’ severity of dry eye was categorized using 2 evaluations: physician assessment and a composite score that combined traditional clinical measures (Schirmer’s test and lissamine green staining) and a symptoms-based measure (patients’ perception of ocular symptoms, as assessed using the McMonnies Dry Eye Questionnaire [36] and the National Eye Institute Visual Functioning Questionnaire [NEI VFQ-25] [37]). Overall mean OSDI scores grouped into “normal”, “mild/moderate”, and “severe” categories were 9.6, 20.8, and 36.3 based on physician assessment, respectively, and 4.5, 18.1, and 36.3 based on patients’ composite score, respectively [28]. In another study by Sullivan et al. [38], a composite score of dry eye severity was established by converting clinical measures (tear osmolarity, Schirmer’s test, TBUT, Meibomian score, corneal and conjunctival staining) and symptoms (from the OSDI) into a common unit system, whereby 0 represented least evidence of the disease and 1 represented most evidence of disease. Based on the composite score, overall mean OSDI scores in patient groups categorized as having “normal”, “mild/moderate”, or “severe” disease were 5.5, 21.0, and 41.2, respectively [38]. Compared with both the Schiffman [28] and Sullivan [38] studies, patients in our study categorized in ITF level 1 had higher overall OSDI scores, and patients in ITF level 2 had similar OSDI scores to patients categorized as having “severe disease” in the study by Schiffman et al. [28]. This suggests that mild-to-moderate dry eye as defined by the ITF guidelines may be associated with a relatively high level of ocular disability compared to other composite signs- and symptoms-based measures of dry eye severity.

Our study is limited by the cross-sectional survey design and lack of patient follow-up. Clinicians were not intentionally masked to patients’ previous dry eye diagnoses and treatments, and hence their assessments of dry eye severity (ITF grading) were subject to possible bias. In addition, no specific method for assessment of conjunctival and corneal staining was stipulated for use at the various study sites, other than clinicians’ standard of care. While the absence of formal grading definitions may have resulted in greater variability, the results are likely to be more reflective of a real-world, clinic-based setting. Despite the inherent variability based on this aspect of the study design, statistical significance was still obtained. On the other hand, as a result of restricting study participation to patients seeking medical consultation for their dry eye symptoms, demonstration of statistical significance may have been facilitated by a selection bias toward homogeneity of the patient sample. Accordingly, the generalizability of our study findings remains to be established using a randomly drawn sample from an unselected dry eye population. Prospective studies with follow-up after initiation of treatment will help further define patients’ perceptions of the impact of dry eye on their overall quality of life.

## Conclusions

Dry eye disease is frequently undertreated [32, 39, 40]. Barriers to appropriate treatment include the time required to adequately diagnose and determine appropriate treatment based on the patient’s severity level, the perception by clinicians that the condition has minor impact on patient well-being, the perception by patients that the disease is normal or less important than other conditions that require treatment, lack of understanding of the disease process [41], and a perceived paucity of therapeutic options [42, 43]. Supplemental artificial tears and eyelid hygiene are common treatment modalities for dry eye disease, yet many patients continue to experience significant disruption to their daily lives. Our findings suggest that dry eye symptoms are frequently troublesome to the patient at all levels of disease severity, and require attention and adequate treatment. A complete and comprehensive medical and ocular history conducted by clinicians should help correctly identify patients who may have ambiguous symptoms without clinical signs or vice versa, and allow proper treatment and management of dry eye disease.

## Abbreviations

ITF: International Task Force
OSDI: Ocular Surface Disease Index
PRO: Patient-reported outcomes
SD: Standard deviation
TBUT: Tear break-up time
VA: Visual acuity
